# Wisconsin

**Published:** 1896-04

**Authors:** W. G. Clark

**Affiliations:** Secretary


### WISCONSIN SOCIETY OF VETERINARY GRADUATES.

THE annual meeting was held in Madison, at the City Hall, Wednesday, February 5, 1896. The meeting was called to order by the President, Dr. J. P. Laws, at 1.45 P.M. The roll was then called, and the following members responded: W. G. Clark, J. R. Kelso, J. P. Laws, G. Ed. Leech, E. L. Morgenroth, E. H. Newton, C. H. Ormond, and J. F. Roub. Visitors: Drs. G. H. Fay, A. H. Hartwig, B. L. Clark, and Walter Pick.

The minutes of the last meeting were read and approved. The President, in his report, gave a brief history of the Association from the time of organization to the present date, which was interesting and instructive. The Secretary's report of accounts was read and accepted. The Treasurer's report was read and accepted.

Dr. S. G. Binger, of Browntown, Green County, was expelled from the Association under charges preferred against him at the last annual meeting.

Dr. Roub read a letter from Dr. Schmitt stating that he had fractured his hip, and could not be present. On motion, the sympathy of the Society was tendered Dr. Schmitt.

Moved by Dr. Leech, and seconded by Dr. Roub, that a resolution of regret in regard to Dr. Brauchle's death be spread upon the minutes; carried.

Applications for membership: Dr. G. H. Fay, Ripon, C.V.C., '93; Dr. A. H. Hartwig, Watertown, C.V.C., '95; Dr. B. L. Clarke, Monticello, C.V.C., '95. A short recess was taken while the Censors considered the applications. The Censors reported favorably on the applications, and it was moved and seconded that they be elected to membership; carried

The Committee on Legislation reported in regard to the bill to regulate the practice of veterinary medicine and surgery which was before the Senate at the last session. The Committee was unable to bring sufficient influence to bear to secure the passage of the bill. On motion, the report was accepted and the Committee discharged.

On motion, the Society proceeded to the election of officers for the ensuing year. The following gentlemen were elected: Dr. G. Ed. Leech Milwaukee, President; Dr. C. H. Ormond, Milwaukee, Vice-President; Dr. W. G. Clark, Beaver Dam, Secretary; Dr. J. P. Laws, Madison, Treasurer; Drs. A. H. Hartwig, Watertown; B. L. Clark, Monticello; and E. H. Newton, Waupun, Censors.

Moved by Dr. Leech, and seconded by Dr. Kelso, that the Society revert to new business; carried.

On motion, the Secretary was instructed to inform the Society of those who have been elected members of the Society and have not qualified. The following names were read and voted upon separately, and dropped from the roll of the Society: Drs. F. E. Stone, S. W. Mount, J. L. Kearney, and I. Philip.

Moved and seconded, that U. S. Inspectors of the Bureau of Animal Industry be invited to attend and take part in the Association meetings.

On motion, the Society adjourned until 7.30 P.M.

*Evening Session.* Meeting called to order by the President at 7.30 o'clock.

The question of legislation was discussed, and it was decided to appoint a committee to draft a bill and present it before the Society at the semi-annual meeting. Drs. Leech, Clark, and Law were appointed as a Committee on Legislation.

On motion, the subject of army veterinary legislation was referred to the Committee on Legislation.

Dr. Leech introduced a resolution requesting that the Association adopt, at its next meeting, as a part of its Code of Ethics, the following: "It shall be deemed a violation of the Code of Ethics for any member of this Association to contract with or through the officers of any live-stock insurance company for the professional treatment of the members' stock so insured; but this rule shall not prevent any member from becoming the examiner of risks, and to act in the capacity of expert for the same. On motion, this was laid over until the next meeting.

Reading of essays: "Shoeing," Dr. E. L. Morgenroth; "Scientific Breeding," Dr. J. R. Kelso; "Equine Relapsing-fever," Dr. T. W. Watson; "Veterinary Meat-inspection by the State, and its Necessity," Dr. G. Ed. Leech; "Professional Ethics," Dr. J. P. Laws. The essays were well prepared, and brought out an interesting discussion. Dr. Watson was unable to attend, and his essay was read by the Secretary.

On motion, it was decided to hold the semi-annual meeting at Milwaukee, subject to the call of the Secretary.

Moved and seconded, that the balance of the special assessment remaining unpaid be remitted.

Adjournment.

W. G. Clark, M.D.C.,
Secretary.

